# Epigenetic regulation during hepatic stellate cell activation

**DOI:** 10.1186/2047-783X-19-S1-S7

**Published:** 2014-06-19

**Authors:** Silke Götze, Eva Quenkert, Claus Kordes, Iris Sawitza, Dieter Häussinger

**Affiliations:** 1 Clinic of Gastroenterology, Hepatology and Infectious Diseases, Heinrich Heine University, 40225 Düsseldorf, Germany

## Background

Hepatic stellate cells (HSC) have recently been identified as liver-resident mesenchymal stem cells and are thought to contribute to liver repair and fibrogenesis [1]. Quiescent HSC are located in the space of Disse and store vitamin A in characteristic lipid droplets. During their activation HSC lose their vitamin A stores and adopt a myofibroblast-like phenotype. This activation of HSC is necessary to enable hepatic differentiation and is accompanied by changes in the expression of many genes such as extracellular matrix protein encoding genes [2]. Some of these genes like Notch1 and Notch3 are regulated by epigenetic mechanisms. Their expression depends on the promoter DNA methylation and is changed during HSC activation [3]. The aim of the present study is a comprehensive analysis of DNA methylation alterations during HSC activation at a global and gene-specific level to elucidate their impact on hepatic stellate cell activation and to identify basic mechanisms of epigenetic control in adult stem cells during differentiation.

## Materials and methods

HSC were isolated from adult male Wistar rats and enriched by density gradient centrifugation (8% Nycodenz) after enzymatic digestion of the liver. Isolated HSC were further purified by vitamin A-dependent *fluorescence-activated cell sorting*. Initially quiescent HSC were activated *in vitro* by culturing the cells on a plastic surface in culture medium containing 10% fetal calf serum. The global DNA methylation of quiescent and activated HSC was analyzed by a 5-methylcytosine (5meC) ELISA (MethylFlash DNA methylation quantification kit, Epigentek) and immunofluorescence staining of methanol-fixed HSC with an antibody against 5meC. Quantitative analysis of repetitive element methylation was performed with a methylation-sensitive restriction followed by a quantitative PCR analysis (qPCR). To identify genes with an altered DNA methylation pattern during HSC activation, a genome-wide DNA methylation analysis was performed (EpiQuest Sequencing, Zymo Research). CpG-rich DNA fragments were concentrated, converted by bisulfite treatment and analyzed by next-generation sequencing to identify 5meC-modified nucleotides. In order to analyze the mechanism of DNA demethylation during HSC activation, the cell proliferation was examined by DNA synthesis determination via BrdU (5-bromo-2'-deoxyuridine) assay and Ki67 Western blot analysis. Furthermore the expression of genes, that were suggested to be important for active DNA demethylation, was investigated by qPCR.

## Results

The analysis of global DNA methylation during *in vitro* activation of HSC by 5meC ELISA revealed a strong decrease of DNA methylation within three days of culture. In contrast, other liver cell types like hepatocytes and Kupffer cells did not show any significant changes in DNA methylation during culture. The decrease in global DNA methylation in cultured HSC was confirmed by immunofluorescence staining with a 5meC specific antibody, which showed a gradual loss of DNA methylation within the first days of culture. Repetitive DNA elements account for approximately half of the genome and are normally highly methylated, thus the observed DNA demethylation may involve repetitive DNA elements. First analysis of identifier elements, which belong to the class of short interspersed nuclear elements (SINE), showed a decrease of DNA methylation during HSC culture. Additional repetitive DNA elements such as Line1 will be analyzed, to determine their contribution to DNA demethylation. The function of global DNA demethylation in adult stem cells are currently unknown, but global DNA demethylation events are under investigation during zygote formation and primordial germ cell migration in early embryogenesis, where they are linked to cellular reprogramming and pluripotency [4]. In order to identify genes, which were regulated by changes in their DNA methylation, a genome-wide DNA methylation analysis was performed in HSC. Next-generation bisulfite sequencing (EpiQuest) was used to compare the DNA methylation between freshly isolated HSC and activated HSC. This analysis identified hyper- as well as hypomethylated genes during HSC activation. It was further investigated, if the DNA demethylation in HSC depends on a passive or an active mechanism. Since passive DNA demethylation occurs during DNA synthesis, a BrdU assay was performed, which showed no DNA synthesis by HSC until the third day of culture. In accordance, Western blot analysis of Ki-67 revealed that HSC were in the G0-phase of the cell cycle until the third day. At that time the DNA demethylation process is almost terminated. Collectively, these data indicated that DNA demethylation in HSC was independent from DNA synthesis and was based on an active mechanism. The molecular mechanism of active DNA demethylation is currently unknown. In order to identify possible components of this DNA demethylation mechanism the expression of potentially involved enzymes during HSC culture are under investigation. The analysis includes enzymes, which are known to play a role in establishing and binding of DNA methylation like Dnmt (DNA methyltransferase) and Mbd (methyl-CpG binding domain protein) or genes, which are suggested to be involved in excision repair mechanisms.

## Conclusions

Our analysis revealed, that the activation of HSC was accompanied by a global DNA demethylation with DNA hyper- and hypomethylation at specific DNA regions, which indicated a strong influence of DNA methylation on HSC activation and differentiation. Furthermore, the DNA demethylation in HSC was based on an active mechanism, which remains to be clarified. Quiescent stellate cells of the liver provide a valuable model system to study mechanisms of active DNA demethylation and to gain new insights in the epigenetic regulation of adult stem cells during their activation and differentiation, which represent important events during liver regeneration and diseases.
